# Application of GRADE: Making evidence-based recommendations about diagnostic tests in clinical practice guidelines

**DOI:** 10.1186/1748-5908-6-62

**Published:** 2011-06-10

**Authors:** Jonathan Hsu, Jan L Brożek, Luigi Terracciano, Julia Kreis, Enrico Compalati, Airton Tetelbom Stein, Alessandro Fiocchi, Holger J Schünemann

**Affiliations:** 1 Department of Clinical Epidemiology and Biostatistics, McMaster University, Hamilton, Ontario, Canada; 2 Department of Medicine, McMaster University, Hamilton, Ontario, Canada; 3 Department of Child Medicine, Fatebenefratelli/Melloni Hospital, Milan, Italy; 4 Department of Epidemiology, Johns Hopkins Bloomberg School of Public Health, Baltimore, Maryland, USA; 5 Allergy and Respiratory Disease Clinic, Department of Internal Medicine, University of Genoa, Genoa, Italy; 6 Department of Public Health UFCSPA, Ulbra and Conceicao Hospital, Porto Alegre, Brazil

## Abstract

**Background:**

Accurate diagnosis is a fundamental aspect of appropriate healthcare. However, clinicians need guidance when implementing diagnostic tests given the number of tests available and resource constraints in healthcare. Practitioners of health often feel compelled to implement recommendations in guidelines, including recommendations about the use of diagnostic tests. However, the understanding about diagnostic tests by guideline panels and the methodology for developing recommendations is far from completely explored. Therefore, we evaluated the factors that guideline developers and users need to consider for the development of implementable recommendations about diagnostic tests.

**Methods:**

Using a critical analysis of the process, we present the results of a case study using the Grading of Recommendations Applicability, Development and Evaluation (GRADE) approach to develop a clinical practice guideline for the diagnosis of Cow Milk Allergy with the World Allergy Organization.

**Results:**

To ensure that guideline panels can develop informed recommendations about diagnostic tests, it appears that more emphasis needs to be placed on group processes, including question formulation, defining patient-important outcomes for diagnostic tests, and summarizing evidence. Explicit consideration of concepts of diagnosis from evidence-based medicine, such as pre-test probability and treatment threshold, is required to facilitate the work of a guideline panel and to formulate implementable recommendations.

**Discussion:**

This case study provides useful guidance for guideline developers and clinicians about what they ought to demand from clinical practice guidelines to facilitate implementation and strengthen confidence in recommendations about diagnostic tests. Applying a structured framework like the GRADE approach with its requirement for transparency in the description of the evidence and factors that influence recommendations facilitates laying out the process and decision factors that are required for the development, interpretation, and implementation of recommendations about diagnostic tests.

## Background

High quality clinical practice guidelines that provide implementable recommendations are the ideal tool to improve patient outcomes in healthcare. Guidelines must, therefore, provide transparent and explicit recommendations accompanied by implementation aids. For example, recommendations about diagnostic tests should consider the downstream consequences of such tests. That is, accurate diagnosis is a prerequisite for successful therapy but an accurate diagnosis should also not be seen in isolation. Establishing a diagnosis does not provide information about whether a patient or a group of patients benefits from the diagnosis. Such benefit should be measured in patient-important outcomes that can include disease-related outcomes (*e.g.*, mortality reduction), psychological consequences of testing as well as resource utilization outcomes. Recommendations about diagnostic tests should consider whether these outcomes, when taken together, achieve net benefit and if this net benefit may be worth the associated resources. However, diagnostic test research rarely focuses on patient important outcomes [1]. Moreover, synthesizing evidence on diagnostic tests is particularly challenging because statistical methods used to aggregate diagnostic accuracy data are conceptually complex, leading to difficulties with the interpretation of results [2]. Despite these challenges, guideline developers make recommendations about the use of diagnostic tests. We believe they all too frequently do so without considering the consequences of applying diagnostic tests in terms of patient important outcomes [3]. In part this may be due to the lack of appropriate guidance for developing recommendations about diagnostic test or strategies.

The consequences of failing to acknowledge all relevant aspects in developing recommendations can be severe. For example, to develop a recommendation about the use of a diagnostic test, one requires either evidence directly comparing alternative diagnostic and management strategies focusing on patient important outcomes, or one must make assumptions about the prevalence, diagnostic test accuracy, efficacy of interventions, and about the prognosis of patients. In prior work of the GRADE working group, we laid out the principles and challenges related to making recommendations about diagnostic tests, but examples for applying GRADE or other explicit and transparent frameworks in these situations are rare [4].

Despite the lack of applying transparent frameworks in the development of recommendations about diagnostic tests, it is likely that healthcare practitioners remain unaware of these limitations and implement guideline recommendations that lack transparency about the assumptions underlying the recommendations, including recommendations about the use of diagnostic tests. Thus, the guideline enterprise requires methods for engaging developers of recommendations in a way that they better understand the consequences of performing diagnostic tests to facilitate implementation of recommendations. These methods include guidance on how to present evidence to guideline developers and healthcare practitioners, moving from evidence to recommendations and then formulating recommendations that facilitate implementation.

Therefore, we describe challenges and solutions related to developing recommendations about diagnostic tests with guideline panels. Our case study is based on using the GRADE approach for a guideline with the World Allergy Organization (WAO) [5] in the clinical area of cow's milk allergy (CMA) that affects 1.9% to 4.9% of infants [6-11]. In this article, we address considerations about specifying patient-important outcomes and summarizing evidence for guideline panels in a comprehensive and structured manner. Furthermore, we describe the group and consensus processes that this guideline panel used to ensure transparent and evidence-based recommendations.

This approach can serve as guidance for panels wishing to implement the GRADE approach, a methodology that has been adopted by over 50 organizations [12], or similar approaches to develop recommendations about diagnostic tests. It should also raise awareness of what guideline users ought to demand from diagnostic recommendations to facilitate the interpretation and strengthen confidence in the recommendations.

## Methods

### General methods

We conducted a case study based on written records, meeting minutes, and critical analysis of the process used to develop the WAO CMA guidelines. Three of the contributors to this article (HJS, JLB and JK) are members of the GRADE working group and have, to a varying degree, contributed to the development of the GRADE approach.

### Panel selection and composition

The panel for this guideline included 22 international members including allergists, paediatricians, gastroenterologists, dermatologists, family physicians, epidemiologists, guideline developers, allergists, food chemists, and representatives of patient organizations. The evidence synthesis and development of clinical recommendations was led by two methodologists (HJS and JLB), who had extensive experience in applying the GRADE approach.

### Conflict of interest

Prior to meeting, panel members were asked to complete written conflict of interest declarations, as recommended by the World Health Organization [13] and American Thoracic Society [14]. The panel agreed that members would recuse themselves or be excused by the chairs from discussion and voting on particular recommendations, if necessary.

### Group process

During the guideline development, a core group met regularly to guide the evidence synthesis. Whenever input from the entire panel was required initially, we solicited it via email and teleconference calls ensuring an economic and streamlined process. A face-to-face meeting of all panel members was held in December 2009 to review the systematically compiled evidence, discuss the recommendations, and agree on their wording and strength. Recommendations that required additional clarification and discussion were finalized during a follow-up conference call.

**Figure 1:**
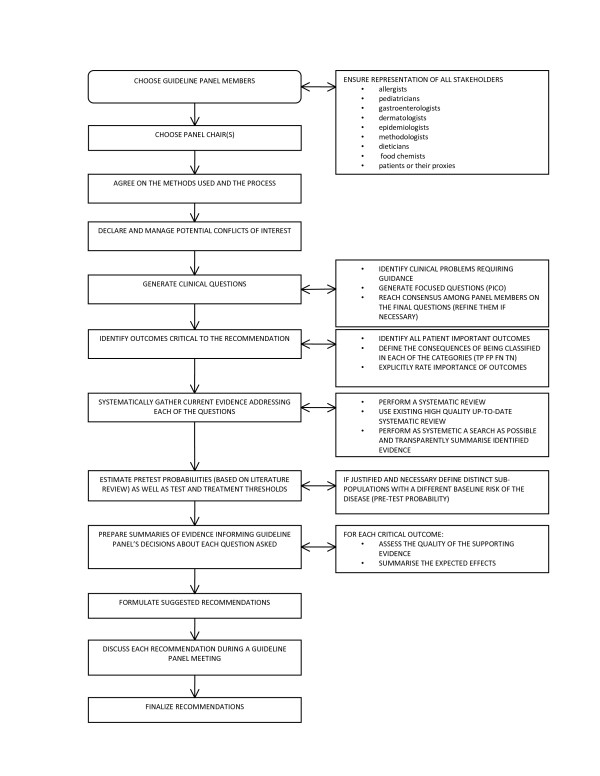
**General process followed for developing clinical practice guideline on diagnostic tests**.

Generally, group processes followed a modified Delphi method prior to the meeting (emailed questions seeking independent decisions with a formal and explicit method of aggregation of responses and feedback) and a structured discussion method during the meeting [15]. This latter method was particularly useful in achieving basic understanding about the complex methodological issues in developing diagnostic recommendations and for building consensus on recommendations. Figure 1 describes the overall process.

### Formulating questions and deciding on the importance of outcomes

It is not unusual for panels to spend one or more meetings on deliberating about what should be covered in a guideline, including developing the healthcare questions of interest. Applying GRADE begins with formulating appropriate clinical questions using the PICO or another structured format [16]. This step leads to focussed clinical questions pertaining to a defined population (P) for whom the diagnostic strategy or intervention (I) is being considered in relation to a comparison strategy (C) according to defined patient outcomes (O).

The CMA panel determined that the population of interest would be patients--adults and children--suspected of IgE-mediated CMA (*i.e.*, those in whom the diagnosis is uncertain). In search for a reference standard, the panel agreed that a blinded oral food challenge (OFC) would be considered a proper reference test (gold standard) in the diagnosis of CMA, against which all test should be evaluated.

An index test (*i.e.*, the test of interest or a 'new' test) can play one of three roles in the existing diagnostic pathway: act as a triage (to minimize use of invasive or expensive tests), replace a current test (to eliminate tests with worse test performance compared to a current test, greater burden, invasiveness, or cost), or add-on (to enhance accuracy of a diagnosis beyond current test) [17]. During the question-generation phase, panel members indicated that they were interested in the index tests as a replacement for the reference standard due to the risks, resource utilization, and burdens associated with performing an OFC.

The main challenge in developing recommendations for diagnostic questions is for panels to understand the implications of the diagnostic test and the quantitative information that diagnostic test accuracy data can provide [4]. GRADE, when making recommendations for diagnosis, provides a structured framework that considers the following outcomes: the patient-important consequences of being classified as true positive (TP), true negative (TN), false positive (FP), or false negative (FN); consequences of inconclusive results; complications of a new test and a reference standard; and resource use (cost). For example, nearly every test inevitably leads to both correctly classified patients (that can be further separated into TP and TN) and incorrectly classified patients (FP and FN). Correct classification is usually associated with benefits or a reduction in adverse outcomes, while incorrect classification is associated with worse consequences (harms), including failure to treat and potentially reduce burden of disease. A guideline panel needs to evaluate whether the benefits of a correct classification (TP and TN) outweigh the potential harms of an incorrect classification (FP and FN). However, the benefits and harms follow from subsequent action and are determined by probabilities of outcome occurrence and the importance of these outcomes to patients (*e.g.*, mortality, morbidity, symptoms, *et al*.). If the benefits of being correctly classified by the test (as TP or TN) are sufficiently greater than harms associated with being incorrectly classified (as FP or FN), the guideline panel may be inclined to accept lower accuracy of a diagnostic test when recommending its use.

For these recommendations, the specific consequences (*i.e.*, what outcomes important to these patients usually happen as a result of subsequent management or lack thereof; these are based on the assumptions about the efficacy of subsequent treatment) for patients of being classified as TP, TN, FP, and FN were first suggested by two clinicians experienced in managing patients with CMA (AF and LT). The patient-important consequences were subsequently refined explicitly by the guideline panel using the Delphi method (see Table 1) and were used to objectively weigh considerations when making recommendations. These clinical implications of correct diagnosis and misclassification (based on assumptions of efficacy of interventions) were provided to panel members who weighed these considerations in making recommendations and judging the importance of outcomes.

**Table 1:** Example of the patient-important consequences of being classified into TP, TN, FP, and FN categories

**Question 1**: Should **skin prick tests **be used for the diagnosis of IgE-mediated CMA in patients suspected of CMA?	
**Population**	Patients suspected of cow's milk allergy (CMA)

**Intervention**:	Skin prick test (SPT)

**Comparison**	Oral food challenge (OFC)

**Outcomes**	

**TP**	The child will undergo OFC, which will turn out positive with risk of anaphylaxis, albeit in controlled environment; burden on time and anxiety for family; exclusion of milk and use of special formulae. Some children with high pre-test probability of disease and/or at high risk of anaphylactic shock during the challenge will not undergo challenge test and be treated with the same consequences of treatment as those who underwent food challenge.

**TN**	The child will receive cow's milk at home with no reaction, no exclusion of milk, no burden on family time and decreased use of resources (no challenge test, no formulae); anxiety in the child and family may depend on the family; looking for other explanation of the symptoms.

**FP**	The patient will undergo an OFC, which will be negative; unnecessary burden on time and anxiety in a family; unnecessary time and resources spent on oral challenge. Some children with high pre-test probability of CMA would not undergo challenge test and would be unnecessarily treated with elimination diet and formula that may led to nutritional deficits (*e.g.*, failure to thrive, rickets, Vit D or calcium deficiency); also stress for the family and unnecessary carrying epinephrine self injector which may be costly as well as delayed diagnosis of the real cause of symptoms.

**FN**	The child will be allowed home and will have an allergic reaction (possibly anaphylactic) to cow's milk at home; high parental anxiety and reluctance to introduce future foods; may lead to multiple exclusion diet. The real cause of symptoms (*i.e.*, CMA) will be missed leading to unnecessary investigations and treatments.

**Inconclusive results**	Either negative positive control or positive negative control: the child would repeat SPT which may be distressing for the child and parent; time spent by a nurse and a repeat clinic appointment would have resource implications; alternatively, child would have sIgE measured or undergo food challenge

**Complications of a test**	SPT can cause discomfort or exacerbation of eczema that can cause distress and parental anxiety; food challenge may cause anaphylaxis and exacerbation of other symptoms.

**Resource utilization (cost)**	SPT adds extra time to clinic appointment however; OFC has much greater resource implications

TP - true positive (being correctly classified as having CMA), TN - true negative (being correctly classified as not having CMA), FP - false positive (being incorrectly classified as having CMA), FN - false negative (being incorrectly classified as not having CMA); these outcomes are always determined in comparison with a reference standard (i.e., food challenge test with cow's milk)

Guideline panel members rated the relative importance of all outcomes, given their associated consequences, on a scale from one (informative but not important for decision making) to nine (critical for decision making) *a priori *(without having seen the summary of the evidence).

### Identifying distinct subgroups of patients at different risk for target condition (pre-test probability)

In formulating a diagnosis, clinicians consider a list of possible target conditions and estimate the probability associated with each of them (*i.e.*, pre-test probability). Depending on this probability, a diagnostic test can be used with an intention to either 'rule in' or to 'rule out' a target condition. However, test accuracy and potential complications of performing it may be such that the test will be useful for one of the two purposes, but not for the other. Thus, in making recommendations about the use of diagnostic tests, one needs to consider groups of patients with different initial (pre-test) probabilities of the target condition.

For the CMA guidelines, the panel decided to make recommendations for patients with low pre-test probability of CMA (*e.g.*, patients with nonspecific gastrointestinal symptoms), those with moderate pre-test probability (*i.e.*, average prevalence of CMA in all studies included in our systematic review), and those with a high pre-test probability (*e.g.*, patients with history of anaphylaxis likely to be caused by cow's milk).

To generate approximate values of pre-test probabilities (high, average, low), we abstracted the prevalence of CMA in comparable populations identified in the studies in our systematic reviews that informed the guidelines. The estimate for high pre-test probability was obtained from populations of patients suspected of CMA with a history of anaphylaxis. The percentage of this high-risk population who actually then were diagnosed with CMA (as verified by the reference standard) was used as the estimate for high pre-test probability. The estimate for low pre-test probability was obtained from the prevalence of CMA in patients suspected of the condition with nonspecific GI symptoms. A third category--an average pre-test probability--was estimated based on the average prevalence of CMA in all studies included in our systematic review. To facilitate understanding and implementation of recommendations, we provided examples of common clinical presentations that clinicians could use to estimate if the individual patient is at high, average, or low initial risk of CMA when corresponding with panel members and for guideline users.

Using this approach, the high pre-test probability was estimated to be approximately 80%, low pre-test probability was estimated to be approximately 10%, and average pre-test probability was estimated to be approximately 40%. These values, in combination with diagnostic accuracy from the systematic review, were used to calculate the number of patients per 1,000 that would be categorized to TP, TN, FP, and FN for each index test depending on pre-test probability.

### Test and treatment threshold work-up

Guideline panel members making recommendations about the use of diagnostic tests must understand that with new information provided by a diagnostic test, the probability of the target condition can increase or decrease. A useful diagnostic test can increase the probability of the target condition across a certain threshold where the physician is confident to start treatment (*i.e.*, treatment threshold). Alternatively, a useful diagnostic test can decrease the probability of the target condition below a certain threshold where the physician is confident to stop testing and rule out the disease (*i.e.*, testing threshold). In other words, diagnostic tests that are of value to clinicians will sufficiently reduce uncertainty about the target condition to rule it in or rule it out.

We solicited the panel members' treatment and testing thresholds for CMA to gauge the level of uncertainty that clinicians are willing to tolerate in ruling CMA in or out while considering the potential consequences. This information would impact the test accuracy required in order for a diagnostic test to be useful in moving the uncertainty above the treatment threshold or below the testing threshold. We asked panel members to estimate treatment and testing thresholds specifying a clinical setting based on history, clinical presentation, and results of index tests alone (*i.e.*, without performing a reference test, the OFC). In detail, we applied the following process. Together with the exercise to determine the importance of outcomes, we invited panel members by email to participate in an 'exercise to attempt to estimate the thresholds at which a clinician stops testing for CMA and either starts treatment (CMA very probable) or informs the patient/parents that CMA is not responsible for the symptoms (CMA very improbable) using four different scenarios (Additional file 1: Appendix 1 includes the detailed exercise).' We informed them that we acknowledge that these thresholds we asked to estimate are subjective and depend on one's values and preferences. We also acknowledged that the four scenarios we presented were a simplification of real life situations but that this may be an acceptable trade off between comprehensiveness and simplicity. Following a detailed description of concepts about test and treatment thresholds, contextualization for CMA, provision of probabilities for outcomes and cost estimates, we asked participants to determine their test and treatment thresholds for four scenarios that were described in detail (see Additional file 1: Appendix 1). We utilized the results of this survey to explore where the thresholds for test recommendations are located along the probabilities of 0 to 100%.

### Preparation of evidence profiles

For each question, we prepared one or more evidence profiles summarising the information about the relevant outcomes. Evidence profiles present a concise summary of estimated effects and an assessment of the quality of supporting evidence to support informed decision making by the panel members. Evidence profiles should ideally be based on a systematic review. Because we did not identify any existing systematic review of the use of tests for the diagnosis of CMA, we performed systematic reviews. We searched MEDLINE, EMBASE, and the Cochrane Library (including Cochrane Central Register of Controlled Trials, DARE, NHS EED) for relevant studies. Studies published up to September 2009 were included. Using the results from the systematic review, we estimated the pooled accuracy of each test. This served as an estimate for the number of patients that would be classified into TP, TN, FP, and FN per 1,000 patients tested. To assess the quality of available evidence, we used the categories described by GRADE [4] and the QUADAS tool [18] relating to the risk of bias, directness of the evidence, consistency and precision of the results, and the likelihood of the publication bias. Based on the different initial probabilities of CMA and the estimated accuracy of each test being evaluated, we calculated the proportion of patients who would be classified as TP, TN, FP, and FN per 1,000 patients tested (see Table 2). Accuracy of the tests was estimated based on a meta-analysis of the review results. Table 3 shows the evidence profile prepared for one of the questions posed in the guidelines.

**Table 2:** Example calculation for determining number of patients classified as TP/TN/FP/FN per 1,000 based on pre-test probability of 20% (based on population with 20% prevalence of CMA in target population)

		Reference standard		
			
		Disease present	Disease absent	
**New Test**	Positive	TP = sensitivity × **200**	FP = (1 - specificity) × **800**	
		
	Negative	FN = (1 - sensitivity) × **200**	TN = specificity × **800**	

Prevalence: **20%**		**200**	**800**	**1000**

**Table 3:** Example of evidence profile generated based on systematic review conducted for these guidelines

**Question 1, Profile 1: Should skin prick tests be used for the diagnosis of IgE-mediated CMA in patients suspected of CMA? Cut-off **≥**3 mm | All populations**										
**Outcome**	**No. of studies**	**Study design**	**Factors that may decrease quality of evidence**					**Final quality**	**Effect per 1000**^**1**^	**Importance**
						
			**Limitations**	**Indirectness**	**Inconsistency**	**Imprecision**	**Reporting bias**			

**True positives **(patients with CMA)	23 studies (2302 patients)	Consecutive or non-consecutive series	Serious^2^	None	Serious^3^	None	Unlikely	⊕⊕OO low	Prev 80%: 536 Prev 40%: 268 Prev 10%: 67	CRITICAL
**True negatives **(patients without CMA)	23 studies (2302 patients)	Consecutive or non-consecutive series	Serious^2^	None	Serious^3^	None	Unlikely	⊕⊕OO low	Prev 80%: 108 Prev 40%: 324 Prev 10%: 486	CRITICAL
**False positives **(patients incorrectly classified as having CMA)	23 studies (2302 patients)	Consecutive or non-consecutive series	Serious^2^	Serious^4^	Serious^3^	None	Unlikely	⊕OOO very low	Prev 80%: 92 Prev 40%: 276 Prev 10%: 414	CRITICAL
**False negatives **(patients incorrectly classified as not having CMA)	23 studies (2302 patients)	Consecutive or non-consecutive series	Serious^2^	None	Serious^3^	None	Unlikely	⊕⊕OO low	Prev 80%: 264 Prev 40%: 132 Prev 10%: 33	CRITICAL
Inconclusive^5^	1 study (310 patients)	Non-consecutive series	-	-	-	-	-	-	-	IMPORTANT
Complications	Not reported	-	-	-	-	-	-	-	-	NOT IMPORTANT
Cost	Not reported	-	-	-	-	-	-	-	-	NOT IMPORTANT

Footnotes 1 to 5 provide detailed rationale underlying ratings.

^1^Based on combined sensitivity of 67% (95% CI: 64 to 70) and specificity of 74% (95% CI: 72 to 77)

^2^Most studies enrolled highly selected patients with atopic eczema or gastrointestinal symptoms, no study reported if an index test or a reference standard were interpreted without knowledge of the results of the other test, but it is very likely that those interpreting results of one test knew the results of the other; all except for one study that reported withdrawals did not explain why patients were withdrawn.

^3^Estimates of sensitivity ranged from 10% to 100%, and specificity from 14% to 100%; we could not explain it by quality of the studies, tests used or included population

^4^There is uncertainty about the consequences for these patients; in some a diagnosis of other potentially serious condition may be delayed

^5^One study in children <12 month of age reported 8% inconclusive challenge tests but did not report number of inconclusive skin prick test

## Results

Descriptive summaries of evidence providing the interpretation of numerical results accompanied evidence profiles. These summaries explicitly stated the results of the literature searches, provided additional information about the included studies, enrolled patients, and tests that had been used. They also described the anticipated benefits and downsides of using an index test relative to a reference standard, additional information that might be relevant for clinical use, and suggested final conclusions about the use of the new test.

During the full panel meeting, members reviewed the evidence summaries, draft guidelines, discussed recommendations, and revised the recommendations if necessary. Consensus was reached on all recommendations and their strength considering the quality of supporting evidence, the balance of desirable and undesirable consequences of using a new test, cost, and patients' values and preferences. No recommendation required voting. The test and treatment threshold workup yielded variable results from guideline panel. Some panel members indicated that they were not willing to accept any residual uncertainty about the presence of CMA. This aversion to any uncertainty is evidenced by providing high treatment thresholds when only the index tests were used. These high treatment thresholds identified a type of clinician who would always perform reference test (OFC). As a consequence, recommendations were made expressing that for settings where OFC would always be performed, index tests would be redundant given their limited accuracy and should not be used.

To increase the transparency and interpretation of recommendations, values, and preferences that panel members assumed when making judgements about the balance of desirable and undesirable consequences of using a new test were explicitly stated with each recommendation. Any additional information that the guideline panel thought might improve the understanding and implementation of the recommendation are provided in the remarks section [5].

Following the GRADE approach, we classified recommendations as either strong or conditional (also known as 'weak'). In total, 15 recommendations about the use of diagnostic tests were made.

## Discussion

Strengths of this approach include consideration of the unique challenges in making recommendations for diagnostic tests. Despite the reliance on modelling assumptions for treatment efficacy, we looked beyond test accuracy to explicitly outline the risks and benefits for patients being classified as TP, TN, FP, or FN, as per the GRADE approach, and were able to engage a panel with a limited experience in the development of guidelines about diagnostic tests in this process [4]. These considerations are otherwise left to the treating clinician. Based on this exercise and the challenges with understanding diagnostic test accuracy data, additional support is required for those making and using recommendations about diagnostic tests. We were able to provide an understanding of the pre-test probability and clinicians' testing/treatment thresholds that allowed the guideline to provide evidence-based recommendations for clinical practice.

Key lessons from the development of these guidelines include an emphasis on concepts related to diagnosis in evidence-based medicine. Panel members had expressed that interpretation of diagnostic accuracy from sensitivity and specificity was cognitively challenging. When we translated these values into number of patients who would be classified as TP, TN, FP, and FN per 1,000 patients tested combined with explicit judgments about the burdens associated with being misclassified, panel members were able to more easily grasp the clinical implications of diagnostic tests under review.

This underscores the importance of presenting evidence for guideline development in standardized format that minimizes cognitive biases and emphasizes patient important outcomes. Our findings are consistent with guideline development literature that suggests that panel members may not be familiar with the methods and processes that are used in developing evidence-based recommendations, especially with a multidisciplinary group of diverse backgrounds [19]. Thus, training and support, whether formal or informal, is key to ensuring understanding and facilitate active participation.

The translation from sensitivity and specificity to clinical implications of the diagnostic tests in the form of number of patients classified as TP, TN, FP, and FN per 1,000 tested was possible because of our pre-test probability workup. Distinguishing between high, average, and low pre-test probabilities also allows clinicians to categorize the spectrum of patients in utilizing the guideline. In addition, these subgroups allow for consideration of particular benefits and risks offered by a new (index) test unique to the subgroup (*e.g.*, lower accuracy of index tests may be acceptable for patients with high pre-test probability when weighed against potential for adverse effects with a reference standard, such as anaphylaxis with OFC).

Soliciting treatment and testing threshold from panel members also had significant implications on the recommendations. The variability in results highlighted the differences among panel members with regards to the level of uncertainty that they were willing to accept in diagnosing CMA. It revealed a group of clinicians who would always perform the reference standard as they would not tolerate the uncertainty left by other tests, regardless of complications or risks associated with OFC. Thus, recommendations offered guidance to this type of clinicians in advising against the use of index tests combined with OFC as they would be redundant given their limited sensitivity and specificity.

## Conclusion

We describe the application of the GRADE approach to the development of diagnostic recommendations. This case study provides useful guidance for guideline developers and clinicians about what they ought to demand from clinical practice guidelines to facilitate implementation and strengthen confidence in recommendations about diagnostic tests. The particular challenges of making diagnostic recommendations were met by developing evidence profiles explicitly defining the patient important outcomes associated with being classified as TP, TN, FP, and FN in addition to providing sensitivity and specificity of diagnostic tests that would determine the number of patients that would fall into each group. Furthermore, there was an emphasis on diagnosis concepts in evidence-based medicine, such as a consideration of pre-test probability and test/treatment thresholds. Throughout the guideline, a structured multidisciplinary panel process ensured the methodological soundness and clinical relevance of recommendations. In summary, applying a structured framework like the GRADE approach with its requirement for transparency in the description of the evidence and factors that influence recommendations facilitates laying out the process and decision factors that are required for the development, interpretation, and implementation of recommendations about diagnostic tests..

## Competing interests

All authors have completed the Unified Competing Interest form at http://www.icmje.org/coi_disclosure.pdf (available on request from the corresponding author) and declare that: no author has relationships with that might have an interest in the submitted work in the previous three years; their spouses, partners, or children have no financial relationships that may be relevant to the submitted work; and JLB and JK are members of the GRADE Working Group, HJS is currently co-chair of the GRADE Working Group. JLB and HJS are co-developers of GRADEpro that is copyrighted by McMaster University. They have not received payments from for-profit organizations that are relevant to the work described in this manuscript. Work on the GRADE Working Group is not compensated. JLB and HJS have supported the dissemination of the GRADE approach worldwide. While the work on the guideline was funded by the World Allergy Organization, the work on this document did not receive specific funding. JH, LT, EC, and AF declare no competing interest.

## Authors' contributions

JH contributed to acquisition, analysis and interpretation of data, and drafting and critical revision of the manuscript. JLB contributed to acquisition, analysis, and interpretation of data, drafting and critical revision of the manuscript, statistical analysis, study design, and study supervision. LT, JK, EC, and AF contributed to acquisition, analysis and interpretation of data, and critical revision of the manuscript. ATS contributed to analysis and interpretation of data and critical revision of the manuscript. HJS conceived of the study, contributed to acquisition, analysis and interpretation of data, drafting, and critical revision of the manuscript, statistical analysis, study design, provided administrative and technical support, supervised the study, and takes responsibility for the content of the manuscript. All authors have read and approved the final manuscript.

## Supplementary Material

Additional file 1 **Appendix 1. Determining test and treatment thresholds**. Click here for file
