# Supplementary material for: Application of GRADE: Making evidence-based recommendations about diagnostic tests in clinical practice guidelines
Source: Implement Sci. 2011 Jun 10;6:62. doi: 10.1186/1748-5908-6-62 (PMC3126717; doi:10.1186/1748-5908-6-62)
Supplement: Additional file 1 — Appendix 1. Determining test and treatment thresholds. [file 1748-5908-6-62-S1.DOC]

**Appendix 1. Determining test and treatment thresholds**

Dear Panel Members,

A guideline recommendation for a diagnostic question depends on the balance between desirable and undesirable consequences for a patient of performing the diagnostic test. Making an accurate diagnosis, *i.e.*, accurately classifying patients as having or not having a given condition, does not necessarily imply that any of the outcomes important to patients (*e.g.*, fewer symptoms, better quality of life) will be affected.

Almost no diagnostic test confirms the presence of a given disease with a 100% certainty. There always is, no matter how small, uncertainty about the correctness of diagnosis. However, during the process of diagnostic investigation there is a point when a clinician becomes confident that the probability of a given disease is high enough to stop further testing and start treatment. For instance, in patients highly suspected of Cow’s Milk Allergy (CMA) (*e.g.*, with a recent history of anaphylactic reaction) and a large wheel positive result of a skin prick test (SPT) one often does not perform a food challenge test, but rather starts treatment based on the clinical presentation and a result of SPT. This decision assumes that the risk of performing a challenge test is too high compared to a risk of making a false diagnosis and starting treatment in an individual who in reality is not allergic to cow’s milk.

Likewise, there is also a point when a clinician becomes confident that the probability of a given disease is low enough to stop further testing without any treatment. For instance, in patients with low suspicion of CMA (*e.g.*, with mild skin lesions and no history of anaphylactic reaction) and a negative result of a SPT one may not perform a food challenge test, but rather stop testing for CMA deciding that based on a clinical presentation and a result of SPT only it is unlikely that CMA is the cause of the symptoms.

In order to be able to make recommendations about using skin SPTs or serum IgE (sIgE) instead of oral food challenge test (OFC) in patients suspected of cow’s milk allergy (CMA), we need to establish these two thresholds of probability of CMA:

1. A probability threshold below which a clinician would be comfortable to stop testing with no resulting treatment based solely on the history, clinical presentation, and the results of SPT or sIgE (without performing OFC). In other words: how unlikely has the diagnosis of CMA to be that one would stop any further testing for CMA and inform a patient and/or family that the patient does not have CMA?

2. A probability threshold above which a clinician would not perform OFC and start treatment based solely on the history, clinical presentation, and the results of SPT or sIgE (without performing OFC). In other words: how likely has the diagnosis of CMA to be before performing OFC that one would start treatment without food challenge?

**Benefits and downsides of OFC test**

For instance, performing OFC reduces the risk that a patient with CMA will continue consuming milk and milk products thereby will continue to experience symptoms with a risk of anaphylactic reactions. It also reduces the risk that a patient without CMA will receive unnecessary elimination diet with exclusion of milk and milk products and may receive a formula treatment which may have cost implications for the family or the healthcare system (if reimbursed).

Food challenges carry a risk of anaphylaxis (estimated to be 10 to 30% of positive reactions in children with pre-test probability of food allergy of <50%) and should be conducted in a supervised medical setting where resuscitation equipment is available.

The risk of complications of a food challenge test depends on the risk of anaphylactic shock during the test. We separate two populations of patients at different risk of shock during OFC test: those at high risk (*e.g.*, with a recent history of severe anaphylactic reaction, history of asthma, multiple allergen sensitization, or very high concentration of sIgE); and those at low risk (*i.e.*, without the above risk factors), who are most likely to develop less severe reactions including mild anaphylaxis

**CMA prognosis**

If left undiagnosed patients will experience the following:

1. Anaphylaxis – 11% (about one-half of them (*i.e.*, ~5% of patients with CMA) will experience mild to severe anaphylactic reaction)
2. Urticaria – 18%
3. Asthma or wheezing – 21%
4. Rhinitis – 20%
5. Colics – 27%
6. Eczema – 35%
7. Vomiting – 48%
8. Diarrhea – 53%

**Benefits and downsides of treatment of CMA**

Older children, adolescents, and adults with CMA who do not consume milk or milk products are at risk of vitamin D and calcium deficiency. Nursing mothers of infants with CMA who avoid milk protein in their diet may need assessment of adequacy of their diet.

Special formulae may be costly. A roughly estimated cost of treatment of one child would be as shown in the table below (please use these rough estimates when considering cost, which may be different in different countries and jurisdictions).

| **Milk/formula** | **Cost per liter**  **[US$ (Euro)]** | **Cost per six months**  **[US$ (Euro)]** |
| --- | --- | --- |
| normal cow’s milk | 1.2 (0.9) | 100 (75) |
| normal formula | 2.5 (2.0) | 230 (160) |
| extensively hydrolysed formula | 9 (6) | 800 (550) |
| soy formula | 7 (5) | 750 (450) |
| rice formula | 9 (6) | 800 (550) |
| amino acid formula | 20 (14) | 1800 (1250) |

**The task**

Please answer the following questions taking into consideration your estimates of the balance between desirable and undesirable consequences of performing or not performing an OFC test in patients suspected of CMA. Please rate it on a scale from 0%, certainty that CMA is absent, to 100% certainty that a patient has CMA.

Please consider the following questions in the context of daily clinical practice not in research setting.

**Testing threshold**

**Q1**

In patients at **low risk** of anaphylactic shock during OFC, at what probability (from 0% to 100%) of the diagnosis of cow’s milk allergy would you stop testing (SPT, sIgE, OFC, *et al*.) and consider CMA unlikely enough to inform the patient/parents that the patient does not have CMA?

A: .......... %

**Q2**

In patients at **high risk** of anaphylactic shock during OFC, at what probability (from 0% to 100%) of the diagnosis of cow’s milk allergy would you stop testing (SPT, sIgE, OFC, *et al*.) and consider CMA unlikely enough to inform the patient/parents that the patient does not have CMA?

A: .......... %

**Treatment threshold**

**Q3**

In patients at **low risk** of anaphylactic shock during OFC, at what probability (from 0% to 100%) of the diagnosis of cow’s milk allergy would you start treatment (elimination diet ± formula) without performing an OFC test with cow’s milk?

A: .......... %

**Q4**

In patients at **high risk** of anaphylactic shock during OFC, at what probability (from 0% to 100%) of the diagnosis of cow’s milk allergy would you start treatment (elimination diet ± formula) without performing an OFC test with cow’s milk?

A: .......... %

If you would like to learn more about the concept of testing and treatment thresholds:


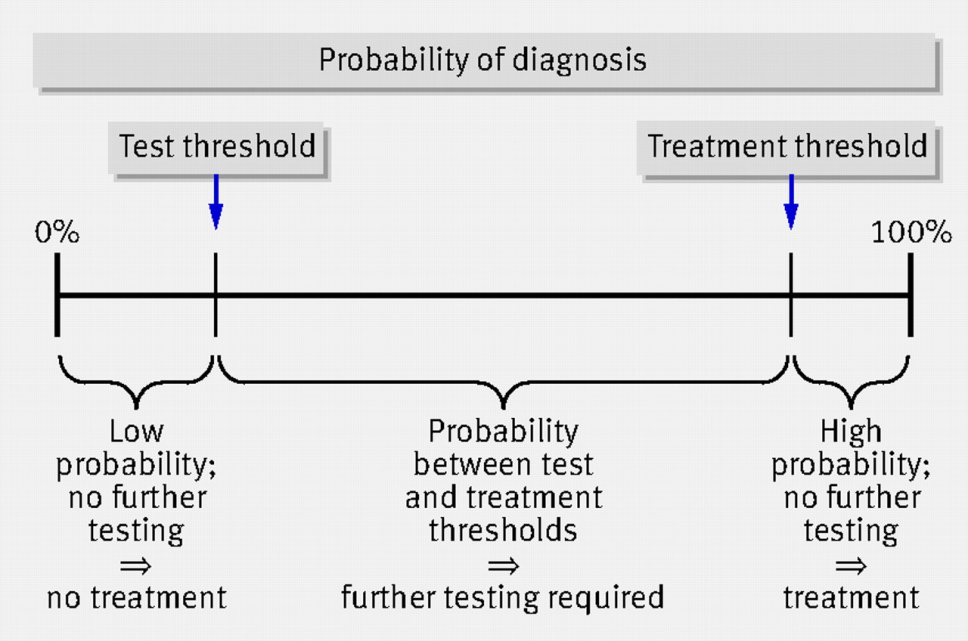


What clinicians expect of a good test is that results change the probability sufficiently to confirm or exclude a diagnosis. Tests, however, are only altering the probability of a disease of interest being present.

If the pre-test probability is below the test threshold, further exclusionary testing would not be useful.

If the pre-test probability is above the treatment threshold, further confirmatory testing that raises the probability further would not be helpful.

When the probability is between the test and treatment thresholds (patients with diagnostic uncertainty), testing will be useful. Test results are of greatest value when they shift the probability across either threshold.

In patients with diagnostic uncertainty (between test and treatment thresholds), if a test result reduces the probability of the condition of interest to below the test threshold, this indicates that the condition is very unlikely, the downsides associated with any further testing and treatment for this condition outweigh any anticipated benefits, and no further testing or treatment for that condition should follow.

If the test result increases the probability of disease to above the treatment threshold, this indicates that the condition is very likely, confirmatory testing that further raises the probability of the condition is unnecessary, and the anticipated benefits of treatment outweigh its potential harms.
